# From the Gut to the Brain and Back: Therapeutic Approaches for the Treatment of Network Dysfunction in Parkinson's Disease

**DOI:** 10.3389/fneur.2020.557928

**Published:** 2020-10-07

**Authors:** Giovanna Paolone

**Affiliations:** Department of Diagnostic and Public Health - Section of Pharmacology, University of Verona, Verona, Italy

**Keywords:** Parkinson's disease, enteric nervous system, dopamine, acetylcholine, postural abnormalities, GDNF

## Abstract

Parkinson's disease (PD) is a complex, multisystem, progressive, degenerative disorder characterized by severe, debilitating motor dysfunction, cognitive impairments, and mood disorders. Although preclinical research has traditionally focused on the motor deficits resulting from the loss of nigrostriatal dopaminergic neurons, up to two thirds of PD patients present separate and distinct behavioral changes. Loss of basal forebrain cholinergic neurons occurs as early as the loss of dopaminergic cells and contributes to the cognitive decline in PD. In addition, attentional deficits can limit posture control and movement efficacy caused by dopaminergic cell loss. Complicating the picture further is intracellular α-synuclein accumulation beginning in the enteric nervous system and diffusing to the substantia nigra through the dorsal motor neurons of the vagus nerve. It seems that α-synuclein's role is that of mediating dopamine synthesis, storage, and release, and its function has not been completely understood. Treating a complex, multistage network disorder, such as PD, likely requires a multipronged approach. Here, we describe a few approaches that could be used alone or perhaps in combination to achieve a greater mosaic of behavioral benefit. These include (1) using encapsulated, genetically modified cells as delivery vehicles for administering neuroprotective trophic factors, such as GDNF, in a direct and sustained means to the brain; (2) immunotherapeutic interventions, such as vaccination or the use of monoclonal antibodies against aggregated, pathological α-synuclein; (3) the continuous infusion of levodopa-carbidopa through an intestinal gel pad to attenuate the loss of dopaminergic function and manage the motor and non-motor complications in PD patients; and (4) specific rehabilitation treatment programs for drug-refractory motor complications.

## Introduction

Mammalian brain activities, from executive and motor functioning to memory and emotional responses, are strictly regulated by the integrity of subcortical projections. Among the subcortical structures, the dopaminergic nigrostriatal pathway and the cholinergic innervations from the basal forebrain play pivotal roles in orchestrating motor and cognitive performance under normal circumstances and in degenerative neurological diseases (1, 2). Research using animal models of Parkinson's disease (PD) has typically focused on the motor deficits resulting from extensive loss of nigrostriatal dopaminergic neurons and on the modeling and treatment of levodopa-induced dyskinesia (3–7). However, up to two thirds of PD patients suffer from a range of non-motor symptoms, including cognitive impairments and mood disorders. Loss of basal forebrain cholinergic neurons occurs as early as the loss of midbrain dopaminergic neurons and likely contributes to the cognitive deficits in PD (8, 9). PD patients also suffer from a propensity for falls, freezing of gait, and associated impairments in posture control and movement efficacy (10) that are not treatable with L-DOPA. These patients have a greater reduction of cortical cholinergic activity relative to PD non-fallers and control subjects (8, 11). Preclinical studies confirm that dual loss in cholinergic and striatal dopamine afferents disrupts posture control and movement efficacy in conditions requiring attention control (12).

In addition to these subcortical changes, increasing evidence suggests that PD pathology can arise in the gut. Clinically, gastrointestinal symptoms often appear in patients before other neurological signs and aggregates of α-synuclein (α-syn) have been found in the enteric nerves of PD patients. The mechanisms through which the disease spreads remain unclear, but it is believed to start in the gut and then move retrogradely to the brain via the vagal nerve or begin in the vagal dorsal motor nucleus and move to the gut in an anterograde way (13–15).

Finally, clinical evaluations found rehabilitation strategies, such as a promising non-drug-based approach able to influence the progression of PD lasting long after the program break, therefore suggesting the involvement of the anatomical substrate accompanying the disease (16, 17).

**Figure 1:**
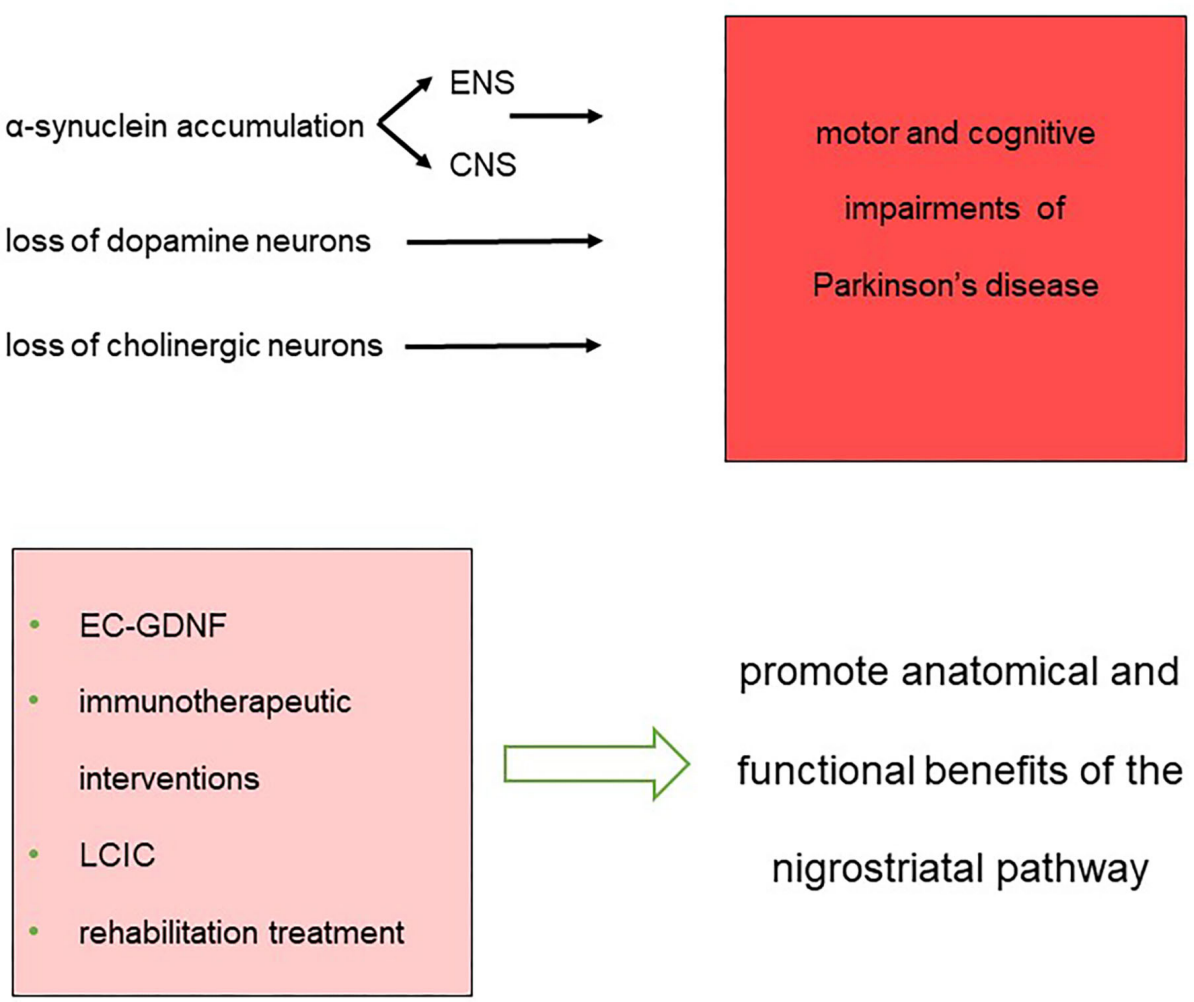
Synopsis of the impairments and therapeutic approaches that constitute the landmarks for the treatment of network dysfunction in Parkinson's disease.

These findings further strain the urge to explore the plastic changes occurring at multiple levels, including cortical and subcortical areas, spinal cord, nerve trucks, and muscles. Understanding the contribution of central and peripheral anatomical rearrangements to the symptoms and recovery could lead to the development of rehabilitation strategies able to counteract the maladaptive changes induced by the disease, ultimately improving patients' quality of life (Figure 1).

## Striatal Microcircuit Alterations and Behavioral Outcomes

The hallmark of PD is the degeneration of dopaminergic neurons in the substantia nigra *pars compacta* (SNpc) and subsequent reduction of striatal projections. The striatum is a primary nucleus of the basal ganglia involved in motor control, goal-directed action, habit learning, and reward-related processes (18–20). It consists of projection neurons with medium spiny neurons (MSNs) representing 90–95% of the local interneuron population (INs). Among them, cholinergic interneurons (ChINs) represent only 1–2% of the population but play a crucial role in sensory integration and movement control. The lessening of dopaminergic striatal innervation leads to a reduction in inhibition of the tonically active ChINs, significantly altering the local microcircuit and contributing to the major motor symptoms of PD. ChINs express both D1 and D2 dopaminergic receptors. Activation of D1 receptors induces glutamate co-release and facilitates acetylcholine (ACh) activity (21, 22) while D2 receptor stimulation decreases ChIN activity by sodium current modulation (23, 24). In a reciprocal role, ACh powerfully modulates DA release from terminals originating in the SNpc via nicotinic ACh receptors (nAChRs) on DA axons (25–28). The activation of muscarinic ACh receptors M2 and M4 that are expressed on somatodendritic and axonal sites exert a more complex action on DA release and related motor and reward-related behaviors (29, 30). Although ChINs have traditionally been considered the principle source of striatal cholinergic innervation (31, 32), additional inputs arising from the peduncolo pontine nucleus (PPN) and laterodorsal tegmental nucleus (LDT) have also been identified (33). Although low in numbers, these ChINs play important roles in controlling motor behavior, and prior to the use of L-DOPA, anticholinergic drugs were used to control motor symptoms (34). Of note, Lozovaya et al. (35, 36) report that a subpopulation of striatal ChINs also co-release GABA. Decreased dopaminergic innervation appears to lead to the failure of the GABAergic function in these dual cholinergic/GABAergic cells augmenting the circuits' cholinergic excitatory component.

Post-mortem human brain histology demonstrates that, when the motor symptoms of PD manifest, ~70% of the SNpc DA cells have degenerated (37) together with a marked loss of choline acetyltransferase (ChAT) expressing neurons in the nucleus basalis of Meynert and penduncolopontine nucleus (PPT) and reduced cortical and striatal cholinergic activity (38). Accordingly, a dual-syndrome hypothesis has emerged in which dopaminergic denervation leads to executive/motor function impairments while cholinergic decline underlies learning and attentional goal-driven deficits and poor performance on cognitive neuropsychological tasks (39, 40). Imbalances in striatal activity of these neurotransmitters may impair the normal induction of synaptic plasticity, altering the processing of routine daily experiences and leading to a plethora of cognitive impairments (41). These observations suggest that, rather than having opposite roles, a cooperative, functional interaction occurs between ACh and DA, and therapies targeting both systems may be effective (42, 43).

## The Gut–Brain Connection

The accumulation of intracellular α-syn is among the major pathological changes associated with neuronal degeneration in PD, including those in the SN and cholinergic cells of the dorsal motor nucleus of the vagus (DMV). α-syn is a 140 amino acid protein that, when misfolded, has the ability to spread from cell to cell in a prion-like manner, leading to an accumulation of α-syn aggregates and formation of oligomers that can progress to fibrils and eventually Lewy bodies. Intracellular accumulation of α-syn likely mediates changes in dopamine synthesis, storage, and release in both the central and enteric nervous systems [C-ENS (44)]. Research into the role of α-syn in PD suggests that several early stage, non-motor symptoms of PD may not originate in the SN. α-syn aggregations evolve in nerve cells of the ENS (13, 14, 45, 46). Shortly thereafter, α-syn deposition seems to involve the anterior olfactory nucleus and the dorsal motor nucleus of the vagal nerve [medulla oblongata (47–51)]. This deposition progresses to affect additional nuclei of the brainstem, the mid and forebrain, and eventually cortical regions. Anatomical studies have identified lesions in the ENS in both non-symptomatic and clinically diagnosed and neuropathologically confirmed cases (45, 52), making the exact role of gut pathology on brain DA denervation unclear. Improvements in techniques that reliably discriminate misfolded, aggregated α-syn from physiological α-syn help clarify the role of this marker for prodromal PD (53).

Brain–gut connectivity and interplay is actually quite deep-rooted in medical history. In 1850, Sydney Whiting (54) in his *Memoirs of a Stomach* wrote, “…and between myself and that individual Mr. Brain, there was established a double set of electrical wires, by which means I could, with the greatest ease and rapidity, tell him all the occurrences of the day as they arrived, and he also could impart to me his own feelings and impressions.” More recently, Kaelberer et al. (55) report that enteroendocrine cells synapse with the vagus and rapidly transduce gut stimulus signals through glutamatergic neurotrasmission. Moreover, catecholamines modulate GI motility by controlling ACh release from motor neurons (56) while the number of DA-positive cells in the meyenteric plexus of patients has been reported to be more than 10 times smaller than in control subjects (57).

## Therapeutic Strategies for the Treatment of PD

Pharmacotherapy with L-DOPA remains a mainstay treatment for PD even though its effectiveness wanes with time. Its use is logically based on the observations of dopaminergic cell loss in patients, but our developing understanding of the multicomponent circuits in PD suggests that multiple treatment avenues might lead to more optimal and longer-lasting efficacy. Some of these avenues are discussed below.

### Neurotrophic Factor Therapy

Delivering trophic factors, such as glial cell line–derived neurotrophic factor (GDNF) to the brain is a potential treatment for PD (58). Although not definitive yet, GDNF may slow or perhaps even reverse the loss of dopaminergic function in PD patients (59, 60). Preclinically, the benefits of GDNF are clear as it prevents the loss of nigral neurons and abnormal motor function that occurs following 6-hydroxydopamine (6-OHDA) lesions in rats (61, 62) and MPTP-lesioned monkeys (63, 64). Direct delivery to the brain is needed for GDNF to be effective, and several approaches are underway to achieve this goal. We have focused on one approach based on implanting GDNF-secreting cells, housed in an immunoprotective membrane, into the brain (65). This approach achieves the goals of selective and long-term delivery to the nigrostriatal system, providing a targeted, continuous, *de novo* synthesized source of high levels of GDNF (66–72). For instance, we recently reported sustained, stable, and selective delivery of high levels of GDNF to the rat striatum implanted with human clonal ARPE-19 cells encapsulated into hollow fiber membranes. Long-term efficacy was evidenced by robust neuroprotection of dopaminergic neurons in the SN and fibers in the striatum in 6-OHDA lesioned rats. In the longest duration studies, GDNF implants produced a significant improvement in motor performance that persisted for over 1 year [62 weeks (73)]. Similarly, impressive distribution of GDNF and positive effects on dopaminergic function were observed when larger, clinical-sized devices were implanted for 3 months into the putamen of Göttingen minipigs. Implantation of GDNF-secreting devices resulted in distribution of GDNF throughout the putamen and caudate that robustly upregulated the expression of tyrosine hydroxylase staining in the regions covered by GDNF diffusion (73, 74). Such an approach may be applicable long term to directly deliver therapeutic molecules, such as GDNF, to the striatum in an attempt to rescue dopaminergic neurons that are otherwise destined to die. GDNF provides substantial anatomical and functional benefits of the nigrostriatal pathway in both rodents and primates, but there is discrepancy in the neuroprotective effects of GDNF in the α-syn models of PD likely related to poor brain penetration or limited distribution within the brain parenchyma (75).

A member of the same neurotrophic factor family as GDNF (76), Neurturin (NTN) has also demonstrated promoting the survival of dopaminergic neurons. The first time an NTN expression construct was tested in an animal model of PD via a lentiviral *in vivo* gene transfer approach was published in 2005 (77). In this study the authors found that NTN enhanced function and protected dopamine neurons similar to GDNF. Similar findings were reported when an adeno-associated virus type 2 (AAV2) encoding human NTN was administered to aged or MPTP-treated monkeys (78). Although NTN delivery by viral gene transfer provides long-term expression and widespread distribution in the target region of the bioactive protein with a single procedure, benefits have not been observed in double-blind trials in PD patients (79, 80). Quantitative immunohistochemical analyses of post-mortem brain sections of patients enrolled in the studies and survived from 1.5 months to 8 and 10 years post-surgery revealed a mild but persistent effect of NTN on nigrostriatal neurons, which seem to modestly amplify over time. Unfortunately, the localized responses were less than what had been assessed in animal models and likely were too weak to induce any clinical improvements (81, 82).

### Immunotherapies Directed Against Pathological α-Syn

Immunotherapies were first conceptualized more than 300 years ago (83) and are being used in clinical applications to significantly improve human health and longevity. In the case of degenerative diseases, the use of monoclonal antibodies or vaccination is a means of treating proteopathies across multiple neural populations (84, 85). In transgenic mice, monoclonal α-syn-specific antibodies increased the degradation of neural and glia accumulation of α-syn, reduced synaptic loss, slowed neurodegeneration, and improved behavioral deficits. At an intracellular level, immunization promotes the clearance of α-syn via the lysosomal pathway (78, 86), whereas in the extracellular space, immunization against α-syn aids microglia in the clearance of the toxic protein, thereby reducing cell-to-cell transmission and local inflammatory response (87, 88). In the PD field, several passive immunization therapies are in preclinical development (89–91), and several others have reached various phases of clinical evaluation (92). One drawback of passive approaches is the necessity for repeated, hospital-based intravenous infusions.

Active immunotherapies elicit a self-produced immune response in the host organism and have the potential advantage of providing long-lasting clearance of the target protein. Recently, Affiris, an Austrian company, completed a Phase I trial with two anti-α-syn vaccines PD01A and PD03A. Although the results from the latter have not yet been published, they have reported about the use of PD01A, which was designed to induce antibodies that selectively target aggregated α-syn with much lower affinity for monomeric forms. Eighty-seven percent of patients (21 out of 24) received all six immunizations across 259 weeks. Over 5 years of follow-up, the authors found that the vaccine was safe and well-tolerated and induced humoral immune responses against pathological α-syn. The Phase I clinical trial of the PD01A vaccine was based on a set of preclinical studies using two different transgenic mouse models (93), and although the study was not powered to assess efficacy, patients treated with PD01A showed stabilized clinical scores compared to a placebo group. The results from this study led to the design and initiation of a Phase II clinical trial (94). Though promising, the most critical drawback for α-syn immunotherapy is the lack of a reliable marker of disease-related proteopathy, and therefore, it becomes difficult to monitor disease progression and establish potential target engagement for anti-α-syn.

### Levodopa–Carbidopa Intestinal Gel Therapy

Administration of oral levodopa–carbidopa is still the most effective drug for PD. However, advanced stage and long-term oral administration leads to disabling motor fluctuations (3, 95) due to pulsatile dopamine release and erratic gastric emptying (96). To overcome these side effects, the European Medicines Agency (May 2011, EU/3/01/035) and the U.S. FDA (2015) approved levodopa–carbidopa intestinal gel (LCIG) for the treatment of advanced idiopathic PD with severe motor fluctuation in patients unresponsive to oral treatment. LCIG is a fluidic carboxymethylcellulose gel suspension containing four parts of L-DOPA to one part carbidopa monohydrate (same as oral formulations) administered into the duodenum through a percutaneous endoscopic gastrostomy tube and portable infusion pump [PEG (97–99)]. Delivery of L-DOPA via infusion achieves more stable plasma levels relative to oral treatment. As a consequence, striatal dopaminergic neurons are stimulated in a sustained manner that reduces the occurrence of “off” periods while increasing the “on” time without dyskinesia. Although there are few large-scale evaluations of the long-term efficacy and safety of LCIG (87, 90, 91), this treatment may have specific benefits on freezing of gait and global axial signs (100–104). Improvements have also been observed on non-motor symptoms, such as sleep/fatigue, urinary and sexual functions, gastrointestinal motility, and cognitive and affective comorbidities (105, 106). Adverse events occur more frequently during the early stages of implantation, but these appear to be related to the surgical procedure and stoma inflammation (107). The contribution of dopaminergic as well as noradrenergic, glutamatergic, and GABAergic pathways provide insights into the intricacy of the PD phenomenology and the development of novel disease-modifying approaches in addition to dopamine-replacing therapies. Nevertheless, evidence-based and experimental therapeutics continues to expand providing cautious optimism for the treatment of patients with PD (108, 109).

### Motor Abnormalities and Physical Activity

Nondrug-based approaches are emerging with the potential to improve cognitive and motor impairments and slow the progression of PD (110–112). The pre-Socratic philosophical belief *mens sana in corpore sano* or “healthy mind in a healthy body” has developed into a vibrant field exploring the possibilities that physical activity might improve cognitive functions as a consequence of hippocampal neurogenesis (113), brain angiogenesis (114), and augmented neurotrophic factors (115). In rodent models, exposure to treadmill or wheel running improves balance, and motor velocity through activity-induced hippocampal upregulation of BDNF (116), or striatal increased dopaminergic neurotransmission (117).

According to the Movement Disorder Society-PD (MDS-PD), the clinical diagnosis of PD focuses on a defined motor syndrome (Parkinsonism) based on bradykinesia, rigidity, and resting tremor. In addition to these symptoms, patients often report posture impairments. Postural abnormalities (PA) belong to the motor axial component in which posture may be affected in its orientation, such as stooped posture, camptocormia, and Pisa syndrome or in its balance component, which implies loss of postural reflex (118, 119). These disabling, drug-refractory motor complications of PD lead to imbalance, fall-related injuries, and generalized pain, ultimately reducing quality of life and increasing hospitalization. PA are poorly improved by L-DOPA, which implies that it is unlikely related to the nigrostriatal dopaminergic denervation. However, Schlenstedt and collaborators (120, 121) found that total, upper, and lateral bending were significantly improved when combined medications and deep brain stimulation (DBS) in the subtalamic nucleus were administered.

Factors related to PA associated with PD suggest two mutually non-exclusive pathophysiological pathways involving central (dystonia, rigidity, proprioceptive disintegration) and peripheral (myopathy and soft tissue changes) mechanisms varying between patients and disease progression (122, 123). Although rehabilitation is fundamental in the management of PD, the current approaches only partially improve postural complications. As motor and non-motor components are involved in the neural control of PA, three main elements are fundamental for effective rehabilitation: active self-correction techniques, stabilization exercises, and functional tasks. Based on this, Tinazzi and collaborators (16, 17, 118) have found that a 4-week trunk-specific rehabilitation program improved passive and active control of the trunk and was maintained at 1-month post-treatment. The benefits of training were evident even when PA were assessed through the Unified Parkinson Disease Rating Scale–motor subscale (122, 124).

## Conclusion

The results from preclinical models and clinical research reveal the importance of investing in innovative therapies for the treatment of PD and other neurological and degenerative diseases. Targeted, continuous, and sustained delivery of drugs at the level of the C/ENS are efficacious, safe, and promising though each still requires improvements to reach a more stable and predictable titration of the delivered drugs. Although the understanding of the beneficial effects of physical activity and general activities that are stimulating for the CNS and motor system is limited, evidence suggests a bidirectional interaction where brain functionality orchestrates the periphery and is deeply modulated by external inputs. A refined understanding of the complexity of normal and dysfunctional networks in PD should lead to improved multifaceted and more optimal treatments.

## Author Contributions

The author confirms being the sole contributor of this work and has approved it for publication.

## Conflict of Interest

The author declares that the research was conducted in the absence of any commercial or financial relationships that could be construed as a potential conflict of interest.

## References

[1] Calabresi P Picconi B Parnetti L Di Filippo M A convergent model for cognitive dysfunctions in Parkinson's disease: the critical dopamine-acetylcholine synaptic balance. Lancet Neurol. (2006) 5:974–83. 10.1016/S1474-4422(06)70600-7 17052664

[2] Aldrin-Kirk P Heuer A Rylander Ottosson D Davidsson M Mattsson B Björklund T. Chemogenetic modulation of cholinergic interneurons reveals their regulating role on the direct and indirect output pathways from the striatum. Neurobiol Dis. (2018) 109(Pt A):148–62. 10.1016/j.nbd.2017.10.010 29037828

[3] Paolone G Brugnoli A Arcuri A Daniela Mercatelli Morari M. Eltoprazine prevents dyskinesias by reducing striatal glutamate and direct pathway neuron activity. Mov Disord. (2015) 30:1728–38. 10.1002/mds.26326 26207892

[4] Zigmond MJ Stricker EM Animal models of parkinsonism using selective neurotoxins: clinical and basic implications. Int Rev Neurobiol. (1989) 31:1–79. 10.1016/S0074-7742(08)60277-9 2689379

[5] Simola N Morelli M Carta AR. The 6-hydroxydopamine model of Parkinson's disease. Neurotox Res. (2007) 11:151–67. 10.1007/BF03033565 17449457

[6] Lundblad M Andersson M Winkler C Kirik D Wierup N Cenci MA. Pharmacological validation of behavioural measures of akinesia and dyskinesia in a rat model of Parkinson's disease. Eur J Neurosci. (2002) 15:120–32. 10.1046/j.0953-816x.2001.01843.x 11860512

[7] Lundblad M Picconi B Lindgren H Cenci MA. A model of L-DOPA-induced dyskinesia in 6-hydroxydopamine lesioned mice: relation to motor and cellular parameters of nigrostriatal function. Neurobiol Dis. (2004) 16:110–23. 10.1016/j.nbd.2004.01.007 15207268

[8] Bohnen NI Albin RL The cholinergic system and Parkinson disease. Behav Brain Res. (2009) 221:564–73. 10.1016/j.bbr.2009.12.048 PMC2888997 20060022

[9] Bohnen NI Grothe MJ Nicola J Ray NJ Müller MLTM Teipel SJ. Recent advances in cholinergic imaging and cognitive decline-Revisiting the cholinergic hypothesis of dementia. Curr Geriatr Rep. (2018) 7:1–11. 10.1007/s13670-018-0234-4 29503795 PMC5831510

[10] Yarnall A Rochester L Burn DJ The interplay of cholinergic function, attention, and falls in Parkinson's disease. Mov Disord. (2011) 26:2496–503. 10.1002/mds.23932 21898597

[11] Bohnen NI Kanel P Müller MLTM. Molecular imaging of the cholinergic system in Parkinson's disease. Int Rev Neurobiol. (2018) 141:211–50. 10.1016/bs.irn.2018.07.027 30314597 PMC6218162

[12] Kucinski A Paolone G Bradshaw M Albin RL Sarter M Attention, movement control, and fall propensity: analysis of multi-system model of Parkinson‘s disease using a novel behavioral test system for the assessment of deficits in the cognitive control of gait, balance and complex movement in rats. J Neurosci. (2013) 33:16522–539. 10.1523/JNEUROSCI.2545-13.2013 24133257 PMC6618519

[13] Braak H Del Tredici K Rüb U de Vos RAI Jansen Steur ENH Braak E Staging of brain pathology related to sporadic Parkinson's disease. Neurobiol Aging. (2003) 24:197–211. 10.1016/S0197-4580(02)00065-9 12498954

[14] Braak H Ghebremedhin E Rüb U Bratzke H Del Tredici K. Stages in the development of Parkinson's disease-related pathology. Cell Tissue Res. (2004) 318:121–34. 10.1007/s00441-004-0956-9 15338272

[15] Kim S Kwon SH Kam TI Panicker N Karuppagounder SS Lee S Transneuronal propagation of pathologic alpha synuclein from the gut to the brain models Parkinson's disease. Neuron. (2019) 103:627–41. 10.1016/j.neuron.2019.05.035 31255487 PMC6706297

[16] Gandolfi M Tinazzi M Magrinelli F Busselli G Dimitrova E Polo N Manganotti P Four-week trunk-specific exercise program decreases forward trunk flexion in Parkinson's disease: a single-blinded, randomized controlled trial. Parkinsonism Relat Disord. (2019) 64:268–74. 10.1016/j.parkreldis.2019.05.006 31097299

[17] Demartini B Bombieri F Goeta D Gambini O Ricciardi L Tinazzi M. A physical therapy programme for functional motor symptoms: a telemedicine pilot study. Parkinsonism Relat Disord. (2019). 10.1016/j.parkreldis.2019.05.004. [Epub ahead of print]. 31078400

[18] Pisani A Centonze D Bernardi G Calabresi P. Striatal synaptic plasticity: implications for motor learning and Parkinson's disease. Mov Disord. (2005) 20:395–402. 10.1002/mds.20394 15719415

[19] Desrochers TM Amemori K Graybiel AM. Habit Learning by naive macaques is marked by response sharpening of striatal neurons representing the cost and outcome of acquired action sequences. Neuron. (2015) 87:853–68. 10.1016/j.neuron.2015.07.019 26291166 PMC4594224

[20] Kravitz AV Kreitzer AC. Striatal mechanisms underlying movement, reinforcement, and punishment. Physiology. (2012) 27:167–77. 10.1152/physiol.00004.2012 22689792 PMC3880226

[21] Cai Y Ford CP. dopamine cells differentially regulate striatal cholinergic transmission across regions through corelease of dopamine and glutamate. Cell Rep. (2018) 25:3148–57.e3. 10.1016/j.celrep.2018.11.053 30540946 PMC6658127

[22] Le Moine C Normand E Bloch B Phenotypical characterization of the rat striatal neurons expressing the d1 dopamine receptor gene. Proc Natl Acad Sci USA. (1991) 88:4205–9. 10.1073/pnas.88.10.4205 1827915 PMC51627

[23] Le Moine C Tison F Bloch B. D2 dopamine receptor gene expression by cholinergic neurons in the rat striatum. Neurosci Lett. (1990) 117:248–52. 10.1016/0304-3940(90)90671-U 2094817

[24] Maurice N Mercer J Chan CS Hernandez-Lopez S Held J Tkatch T. D2 dopamine receptor-mediated modulation of voltage-dependent na+ channels reduces autonomous activity in striatal cholinergic interneurons. J Neurosci. (2004) 24:10289–301. 10.1523/JNEUROSCI.2155-04.2004 15548642 PMC6730305

[25] Jones IW Bolam JP Wonnacott S. Presynaptic localisation of the nicotinic acetylcholine receptor beta2 subunit immunoreactivity in rat nigrostriatal dopaminergic neurones. J Comp Neur. (2001) 439:235–47. 10.1002/cne.1345 11596051

[26] Zhou FM Liang Y Dani JA Endogenous nicotinic cholinergic activity regulates dopamine release in the striatum. Nat Neurosci. (2001) 4:1224–9. 10.1038/nn769 11713470

[27] Rice ME Cragg SJ. Nicotine amplifies reward-related dopamine signals in striatum. Nat Neurosci. (2004) 7:583–4. 10.1038/nn1244 15146188

[28] Cachope R Mateo Y Mathur BN Irving J Wang HL Morales M. Selective activation of cholinergic interneurons enhances accumbal phasic dopamine release: setting the tone for reward processing. Cell Rep. (2012) 2:33–41. 10.1016/j.celrep.2012.05.011 22840394 PMC3408582

[29] Moehle MS Conn PJ. Roles of the M4 acetylcholine receptor in the basal ganglia and the treatment of movement disorders. Mov Disord. (2019) 34:1089–99. 10.1002/mds.27740 31211471 PMC6699902

[30] Ztaou S Maurice N Camon J Guiraudie-Capraz G Kerkerian-Le Goff L Beurrier C. Involvement of striatal cholinergic interneurons and m1 and m4 muscarinic receptors in motor symptoms of Parkinson's disease. J Neurosci. (2016) 36:9161–72. 10.1523/JNEUROSCI.0873-16.2016 27581457 PMC6601910

[31] Brimblecombe KR Threlfell S Dautan D Kosillo P Mena-Segovia J Cragg SJ. Targeted activation of cholinergic interneurons accounts for the modulation of dopamine by striatal nicotinic receptors. eNeuro. (2018) 5:ENEURO.0397-17.2018. 10.1523/ENEURO.0397-17.2018 30406189 PMC6220583

[32] Dautan D Huerta-Ocampo I Witten IB Deisseroth KJ Bolam JP Gerdjikov T. A major external source of cholinergic innervation of the striatum and nucleus accumbens originates in the brainstem. J Neurosci. (2014) 34:4509–18. 10.1523/JNEUROSCI.5071-13.2014 24671996 PMC3965779

[33] Kim K Müller MLTM Bohnen NI Sarter M Lustig C. Thalamic cholinergic innervation makes a specific bottom-up contribution to signal detection: evidence from Parkinson's disease patients with defined cholinergic losses. Neuroimage. (2017) 149:295–304. 10.1016/j.neuroimage.2017.02.006 28167350 PMC5386784

[34] Calabresi P Galletti F Saggese E Ghiglieri V Picconi B. Neuronal networks and synaptic plasticity in Parkinson's disease: beyond motor deficits. Parkinsonism Relat Disord. (2007) 13 (Suppl. 3):S259–62. 10.1016/S1353-8020(08)70013-0 18267247

[35] Lozovaya N Eftekhari S Cloarec R Gouty-Colomer LA Dufour A Riffault B. GABAergic inhibition in dual-transmission cholinergic and GABAergic striatal interneurons is abolished in Parkinson disease. Nat Commun. (2018) 9:1422. 10.1038/s41467-018-03802-y 29651049 PMC5897332

[36] Lozovaya N Ben-Ari Y Hammond C Striatal dual cholinergic /GABAergic transmission in Parkinson disease: friends or foes? Cell Stress. (2018) 2:147–9. 10.15698/cst2018.06.142 31225481 PMC6551683

[37] Hornykiewicz O Brain monoamines and Parkinsonism. Natl Inst Drug Abuse Res Monogr Ser. (1975) 3:13–21. 10.1037/e472122004-001 787796

[38] Nakano I Hirano A Parkinson's disease: neuron loss in the nucleus basalis without concomitant Alzheimer's disease. Ann Neurol. (1984) 15:415–8. 10.1002/ana.410150503 6732189

[39] Blatt J Vellage A Baier B Müller NG. The contribution of acetylcholine and dopamine to subprocesses of visual working memory – what patients with amnestic mild cognitive impairment and Parkinson's disease can tell us. Neuropsychologia. (2014) 61:89–95. 10.1016/j.neuropsychologia.2014.06.013 24952112

[40] Kim K Bohnen NI Müller MLTM Lustig C. Compensatory dopaminergic-cholinergic interactions in conflict processing: evidence from patients with Parkinson's disease. Neuroimage. (2019) 190:94–106. 10.1016/j.neuroimage.2018.01.021 29337277 PMC6041186

[41] Calabresi P Maj R Pisani A Mercuri NB Bernardi G. Long-term synaptic depression in the striatum: physiological and pharmacological characterization. J Neurosci. (1992) 12:4224–33. 10.1523/JNEUROSCI.12-11-04224.1992 1359031 PMC6576009

[42] Koshy Cherian A Kucinski A Wu R de Jong IEM Sarter M Co-treatment with rivastigmine and idalopirdine reduces the propensity for falls in a rat model of falls in Parkinson's disease. Psychopharmacology. (2019) 236:1701–15. 10.1007/s00213-018-5150-y 30607479

[43] van Laar T de Deyn PP Aarsland D Barone P Galvin JE Effects of cholinesterase inhibitors in Parkinson's disease dementia: a review of clinical data. CNS Neurosci Ther. (2010) 17:428–41. 10.1111/j.1755-5949.2010.00166.x 21951368 PMC6493905

[44] Goedert M. Neurodegeneration. Alzheimer's and Parkinson's diseases: the The prion concept in relation to assembled Aβ, tau, and α-synuclein. Science. (2015) 349:1255555. 10.1126/science.1255555 26250687

[45] Braak H de Vos RA Bohl J Del Tredici K. Gastric alpha-synuclein immunoreactive inclusions in Meissner's and Auerbach's plexuses in cases staged for Parkinson's disease-related brain pathology. Neurosci Lett. (2006) 396:67–72. 10.1016/j.neulet.2005.11.012 16330147

[46] Garrido-Gil P Rodriguez-Perez AI Dominguez-Meijide A Guerra MJ Labandeira-Garcia JL. Bidirectional neural interaction between central dopaminergic and gut lesions in Parkinson's disease models. Mol Neurobiol. (2018) 55:7297–316. 10.1007/s12035-018-0937-8 29404956

[47] Spillantini MG Schmidt MìL Lee VMY Trojanowski JQ Jakes R Goedert M. Alpha-synuclein in lewy bodies. Nature. (1997) 388:2045–7. 10.1038/42166 9278044

[48] Tillerson JL Caudle WM Parent JM Gong C Schallert T Miller GW Olfactory discrimination deficits in mice lacking the dopamine transporter or the D2 dopamine receptor. Behav Brain Res. (2006) 172:97–105. 10.1016/j.bbr.2006.04.025 16765459

[49] Kuusisto E Parkkinen L Alafuzoff I. Morphogenesis of Lewy bodies: dissimilar incorporation of -synuclein, ubiquitin, and p62. J Neuropathol Exp Neurol. (2003) 62:1241–53. 10.1093/jnen/62.12.1241 14692700

[50] Lang AE Obeso JA. Challenges in Parkinson's disease: restoration of the nigrostriatal dopamine system is not enough. Lancet Neurol. (2004) 3:309–16. 10.1016/S1474-4422(04)00740-9 15099546

[51] Ulusoy A Phillips RJ Helwig M Klinkenberg M Powley TL Di Monte A. Brain-to-stomach transfer of alpha-synuclein via vagal preganglionic projections. Acta Neuropathol. (2017) 133:381–93. 10.1007/s00401-016-1661-y 28012041 PMC5326583

[52] Braak H Rub U Gai WP Del Tredici R. Idiopathic Parkinson's disease: possible routes by which vulnerable neuronal types may be subject to neuroinvasion by an unknown pathogen. J Neural Trans. (2003) 110:517–36. 10.1007/s00702-002-0808-2 12721813

[53] Corbille AG Letournel F Kordower JH Lee J Shanes E Neunlist M. Evaluation of alpha-synuclein immunohistochemical methods for the detection of Lewy-type synucleinopathy in gastrointestinal biopsies. Acta Neuropathol Commun. (2016) 4:35. 10.1186/s40478-016-0305-8 27044604 PMC4820972

[54] Sydney S Momoires of a Stomach. Bedford, MA: Applewood Books (1850).

[55] Kaelberer MM Buchanan KL Klein ME Barth BB Montoya MM Shen X. A gut-brain neural circuit for nutrient sensory transduction. Science. (2018) 361:eaat5236. 10.1126/science.aat5236 30237325 PMC6417812

[56] Schneider SA Boettner M Alexoudi A Zorenkov D Deuschl G Wedel T. Can we use peripheral tissue biopsies to diagnose Parkinson's disease? A review of the literature. Eur J Neurol. (2016) 23:247–61. 10.1111/ene.12753 26100920

[57] Singaram C Ashraf W Gaumnitz EA Torbey C Sengupta A Pfeiffer R. Dopaminergic defect of enteric nervous system in Parkinson's disease patients with chronic constipation. Lancet. (1995) 346:861–4. 10.1016/S0140-6736(95)92707-7 7564669

[58] Choi-Lundberg DL Lin Q Chang YN Chiang YL Hay CM Mohajeri H. Dopaminergic neurons protected from degeneration by GDNF gene therapy. Science. (1997) 275:838–41. 10.1126/science.275.5301.838 9012352

[59] Whone A Luz M Boca M Woolley M Mooney L Dharia S. Randomized trial of intermittent intraputamenal glial cell line-derived neurotrophic factor in Parkinson's disease. Brain. (2019) 142:512–25. 10.1093/brain/awz023 30808022 PMC6391602

[60] Kirkeby K Barker RA. Parkinson disease and growth factors – is GDNF good enough? Nat Rev Neurol. (2019) 15:312–4. 10.1038/s41582-019-0180-6 30948845

[61] Wang L Muramatsu S Lu Y Ikeguchi K Fujimoto K Okada T. Delayed delivery of AAV GDNF prevents nigral neurodegeneration and promotes functionalrecovery in a rat model of Parkinson's disease. Gene Ther. (2002) 9:381–9. 10.1038/sj.gt.3301682 11960314

[62] Dowd E Monville C Torres EM Wong LF Azzouz M Mazarakis ND. Lentivector-mediated delivery of GDNF protects complex motor functions relevant to human Parkinsonism in a rat lesion model. Eur J Neurosci. (2005) 22:2587–95. 10.1111/j.1460-9568.2005.04414.x 16307601

[63] Kordower JH Emborg ME Bloch J Ma SY Chu Y Leventhal L Neurodegeneration prevented by lentiviral delivery of GDNF in primate models of Parkinson's disease. Science. (2000) 290:767–73. 10.1126/science.290.5492.767 11052933

[64] Palfi S Leventhal L Chu Y Ma SY Emborg M Bakay R. Lentivirally delivered glial cell line-derived neurotrophic factor increases the number of striatal dopaminergic neurons in primate models of nigrostriatal degeneration. J Neurosci. (2002) 22:4942–54. 10.1523/JNEUROSCI.22-12-04942.2002 12077191 PMC6757756

[65] Lindvall O Wahlberg LU. Encapsulated cell biodelivery of GDNF: a novel clinical strategy for neuroprotection in Parkinson's disease? Exp Neurol. (2008) 209:82–8. 10.1016/j.expneurol.2007.08.019 17963752

[66] Emerich DF Orive G Thanos C Tornoe J Wahlberg LU. Encapsulated cell therapy for neurodegenerative diseases: from promise to product. Adv Drug Deliv Rev. (2014) 67–68:131–41. 10.1016/j.addr.2013.07.008 23880505

[67] Orive G Santos E Poncelet D Hernández RM Pedraz JL Wahlberg LU. Cell encapsulation: technical and clinical advances. Trends Pharmacol Sci. (2015) 36:537–46. 10.1016/j.tips.2015.05.003 26067102

[68] Simonato M Falcicchia C Paolone G Cell therapy for epilepsy. In: Emerich DF, Orive G, editors. Cell Therapy. New York, NY: Springer Nature (2017) p. 85–98. 10.1007/978-3-319-57153-9

[69] Falcicchia C Paolone G Emerich DF Lovisari F Bell W Fradet T. Seizure-suppressant and neuroprotective effects of encapsulated BDNF-producing cells in a rat model of temporal lobe epilepsy. Mol Ther Methods Clin Dev. (2018) 9:211–24. 10.1016/j.omtm.2018.03.001 29766029 PMC5948312

[70] Emerich DF Kordower JH Chu Y Thanos C Bintz B Paolone G. Widespread striatal delivery of gdnf from encapsulated cells prevents the anatomical and functional consequences of excitotoxicity. Neural Plast. (2019) 11:6286197. 10.1155/2019/6286197 30984255 PMC6432730

[71] Paolone G Falcicchia C Lovisari F Kokaia M Bell W Fradet T. Long-term, targeted delivery of GDNF from encapsulated cells is neuroprotective and reduces seizures in the pilocarpine model of epilepsy. J Neurosci. (2019) 39:2144–56. 10.1523/JNEUROSCI.0435-18.2018 30665947 PMC6507083

[72] Wahlberg LU Lind G Almqvist PM Kusk P Tornøe J Juliusson B. Targeted delivery of nerve growth factor via encapsulated cell biodelivery in Alzheimer disease: a technology platform for restorative neurosurgery. J Neurosurg. (2012) 117:340–7. 10.3171/2012.2.JNS11714 22655593

[73] Wahlberg LU Emerich DF Kordower JH Bell W Fradet T Paolone G Long-term, stable, targeted biodelivery and efficacy of gdnf from encapsulated cells in the rat and goettingen miniature pig brain. Curr Res Pharm. (2020) 1:19–29. 10.1016/j.crphar.2020.04.001 PMC8663965 34909639

[74] Danielsen EH Cumming P Andersen F Bender D Brevig T Falborg L. The DaNeX study of embryonic mesencephalic, dopaminergic tissue grafted to a minipig model of Parkinson's disease: preliminary findings of effect of MPTP poisoning on striatal dopaminergic markers. Cell Transplant. (2000) 9:247–59. 10.1177/096368970000900210 10811397

[75] Decressac M Mattsson B Björklund A Comparison of the behavioral and histological characteristics of the 6-OHDA and α-synuclein rat models of Parkinson's disease. Exp Neurol. (2012) 235:306–15. 10.1016/j.expneurol.2012.02.012 22394547

[76] Airaksinen MS Saarma M. The GDNF family: signalling, biological functions and therapeutic value. Nat Rev Neurosci. (2002) 3:383–94. 10.1038/nrn812 11988777

[77] Fjord-Larsen L Johansen JL Kusk P Tornøe J Grønborg M Rosenblad C. Efficient *in vivo* protection of nigral dopaminergic neurons by lentiviral gene transfer of a modified Neurturin construct. Exp Neurol. (2005) 195:49–60. 10.1016/j.expneurol.2005.03.006 15919076

[78] Herzog CD Dass B Holden JE Stansell J Gasmi M Tuszynski MH Striatal delivery of CERE-120, an AAV2 vector encoding human neurturin, enhances activity of the dopaminergic nigrostriatal system in aged monkeys. Mov Disord. (2007) 22:1124–32. 10.1002/mds.21503 17443702

[79] Marks W Bartus R Siffert J Davis CS Lozano A Boulis N. Double-blind, sham-surgery controlled trial of gene delivery of AAV2-neurturin for Parkinson's disease. Lancet Neurol. (2010) 9:1164–72. 10.1016/S1474-4422(10)70254-4 20970382

[80] Warren Olanow C Bartus RT Baumann TL Factor S Boulis N Stacy M Gene delivery of neurturin to putamen and substantia nigra in parkinson disease: a double-blind, randomized, controlled trial. Ann Neurol. (2015) 78:248–57. 10.1002/ana.24436 26061140

[81] Bartus RT Kordower JH Johnson EM Jr Brown L Kruegel BR Chu Y. Post-mortem assessment of the short and long-term effects of the trophic factor neurturin in patients with α-synucleinopathies. Neurobiol Dis. (2015) 78:162–71. 10.1016/j.nbd.2015.03.023 25841760

[82] Chu Y Bartus RT Manfredsson FP Olanow CW Kordower JH. Long-term post-mortem studies following neurturin gene therapy in patients with advanced Parkinson's disease. Brain. (2020) 143:960–75. 10.1093/brain/awaa020 32203581 PMC7089653

[83] Plotkin S. History of vaccination. Proc Natl Acad Sci USA. (2014) 111:12283–7. 10.1073/pnas.1400472111 25136134 PMC4151719

[84] Baecher-Allan C Kaskow BJ Weiner HL. Multiple sclerosis: mechanisms and immunotherapy. Neuron. (2018) 97:742–68. 10.1016/j.neuron.2018.01.021 29470968

[85] Selkoe DJ. Alzheimer disease and aducanumab: adjusting our approach. Nat Rev Neurol. (2019) 15:365–6. 10.1038/s41582-019-0205-1 31138932

[86] Chatterjee D Kordower JH. Immunotherapy in Parkinson's disease: current status and future directions. Neurobiol Dis. (2019) 132:104587. 10.1016/j.nbd.2019.104587 31454546

[87] Bae EJ Lee HJ Rockenstein E Ho DH Park EB Yang NY. Antibody-aided clearance of extracellular alpha-synuclein prevents cell-to-cell aggregate transmission. J Neurosci. (2012) 32:13454–69. 10.1523/JNEUROSCI.1292-12.2012 23015436 PMC3752153

[88] Masliah E Rockenstein E Adame A Alford M Crews L Hashimoto M. Effects of alpha-synuclein immunization in a mouse model of Parkinson's disease. Neuron. (2005) 46:857–68. 10.1016/j.neuron.2005.05.010 15953415

[89] Masliah E Rockenstein E Mante M Crews L Spencer B Adame A. Passive immunization reduces behavioral and neuropathological deficits in an alpha-synuclein transgenic model of Lewy body disease. PLoS ONE. (2011) 6:e19338. 10.1371/journal.pone.0019338 21559417 PMC3084838

[90] Kim C Spencer B Rockenstein E Yamakado H Mante M Adame A. Immunotherapy targeting toll-like receptor 2 alleviates neurodegeneration in models of synucleinopathy by modulating alpha-synuclein transmission and neuroinflammation. Mol Neurodegener. (2018) 13:43. 10.1186/s13024-018-0276-2 30092810 PMC6085656

[91] Weihofen A Liu Y Arndt JW Huy C Quan C Smith BA Development of an aggregate-selective, human-derived alpha-synuclein antibody BIIB054 that ameliorates disease phenotypes in Parkinson's disease models. Neurobiol Dis. (2019) 124:276–88. 10.1016/j.nbd.2018.10.016 30381260

[92] Jankovic J Goodman I Safirstein B Marmon TK Schenk DB Koller M. Safety and tolerability of multiple ascending doses of PRX002/RG7935, an anti-alpha-synuclein monoclonal antibody, in patients with Parkinson disease: a randomized clinical trial. JAMA Neurol. (2018) 75:1206–14. 10.1001/jamaneurol.2018.1487 29913017 PMC6233845

[93] Mandler M Valera E Rockenstein E Weninger H Patrick C Adame A. Next-generation active immunization approach for synucleinopathies: implications for Parkinson's disease clinical trials. Acta Neuropathol. (2014) 127:861–79. 10.1007/s00401-014-1256-4 24525765 PMC4034750

[94] Volc D Poewe W Kutzelnigg A Lührs P Thun-Hohenstein C Schneeberger A. Safety and immunogenicity of the α-synuclein active immunotherapeutic PD01A in patients with Parkinson's disease: a randomised, single-blinded, phase 1 trial. Lancet Neurol. (2020) 19:591–600. 10.1016/S1474-4422(20)30136-8 32562684

[95] Cenci MA Riggare S Pahwa R Eidelberg D Hauser RA. Dyskinesia matters. Mov Disord. (2020) 35:392–96. 10.1002/mds.27959 31872501

[96] Shoulson I Glaubiger GA Chase TN. On-off response. Clinical and biochemical correlations during oral and intravenous levodopa administration in parkinsonian patients. Neurology. (1975) 25:1144–8. 10.1212/WNL.25.12.1144 812004

[97] Poewe W Antonini A. Novel formulations and modes of delivery of levodopa. Mov Disord. (2015) 30:114–20. 10.1002/mds.26078 25476691

[98] Epstein M Johnson DA Hawes R Schmulewitz N vanagunas AD Gossen ER. Long-term PEG-J tube safety in patients with advanced Parkinson's disease. Clin Transl Gastroenterol. (2016) 7:e159. 10.1038/ctg.2016.19 27030949 PMC4822096

[99] Amjad F Bhatti D Davis TL Oguh O. current practices for outpatient initiation of levodopa-carbidopa intestinal gel for management of advanced Parkinson's disease in the United States. Adv Ther. (2019) 36:2233–46. 10.1007/s12325-019-01014-4 31278691 PMC6822848

[100] Poewe W Bergmann L Kukreja P Robieson WZ Antonini A. Levodopa-carbidopa Intestinal gel monotherapy: GLORIA registry demographics, efficacy, and safety. J Parkinson's Dis. (2019) 9:531–41. 10.3233/JPD-191605 31282424 PMC6700622

[101] Antonini A Marano P Gusmaroli G Modugno N Pacchetti C Sensi M. Long-term effectiveness of levodopa-carbidopa intestinal gel on motor and non-motor symptoms in advanced Parkinson's disease: results of the Italian GLORIA patient population. Neurol Sci. (2020) 41:2929–37. 10.1007/s10072-020-04401-w 32342325 PMC7479015

[102] Richter D Bartig D Jost W Jörges C Stumpe B Gold R. Dynamics of device-based treatments for Parkinson's disease in Germany from 2010 to 2017: application of continuous subcutaneous apomorphine, levodopa-carbidopa intestinal gel, and deep brain stimulation. J Neural Transm. (2019) 126:879–88. 10.1007/s00702-019-02034-8 31222604

[103] Zibetti M Angrisano S Dematteis F Artusi CA Romagnolo A Merola A. Effects of intestinal levodopa infusion on freezing of gait in Parkinson disease. J Neurol Sci. (2018) 385:105–8. 10.1016/j.jns.2017.12.012 29406886

[104] Rispoli V Golfre Andreasi N Penna G Preda F Contini E Sensi M Levodopa/carbidopa intestinal gel infusion therapy: focus on gait and balance. Mov Disord Clin Pract. (2018) 5:542–45. 10.1002/mdc3.12640 30515445 PMC6207104

[105] Modugno N Marano P Sensi M Meco G Solla P Gusmaroli G. Motor and non-motor outcomes in patients with advanced Parkinson's disease treated with levodopa/carbidopa intestinal gel: final results of the GREENFIELD observational study. J Neurol. (2019) 266:2164–76. 10.1007/s00415-019-09337-6 31134377 PMC6687881

[106] Kulisevsky J Bejr-Kasem H Martinez-Horta S Horta-Barba A Pascual-Sedano B Campolongo A. Subclinical affective and cognitive fluctuations in Parkinson's disease: a randomized double-blind double-dummy study of oral vs intrajejunal levodopa. J Neurol. (2020). 10.1007/s00415-020-10018-y. [Epub ahead of print]. 32607644

[107] Petzinger GM Walsh JP Akopian G Hogg E Abernathy A Arevalo P Clinical implications of gastric complications on levodopa treatment in Parkinson's disease. Parkinsonism Relat Disord. (2020) 11:S1353–8020(20)30110–3. 10.1016/j.parkreldis.2020.05.001 32461054

[108] Fox SH Katzenschlager R Lim SH Barton B de Bie RMA Klaus Seppi K. International Parkinson and movement disorder society evidence-based medicine review: update on treatments for the motor symptoms of Parkinson's disease. Mov Disord. (2018) 33:1248–66. 10.1002/mds.27372 29570866

[109] Feustel AC MacPherson A Fergusson DA Kieburtz K Kimmelman J. Risks and benefits of unapproved disease-modifying treatments for neurodegenerative disease. Neurology. (2020) 94:e1–14. 10.1212/WNL.0000000000008699 31792092 PMC7011691

[110] Nithianantharajah J Hannan AJ. Enriched environments, experience-dependent plasticity and disorders of the nervous system. Nat Rev Neurosci. (2006) 7:697–709. 10.1038/nrn1970 16924259

[111] Pang TYC Hannan AJ. Enhancement of cognitive function in models of brain disease through environmental enrichment and physical activity. Neuropharmacology. (2013) 64:515–28. 10.1016/j.neuropharm.2012.06.029 22766390

[112] Hilario WF Laschuk Herlinger A Bianchine Areal L de Moraes SL Alarcon Ferreira T Servane Andrade TE. Cholinergic and dopaminergic alterations in nigrostriatal neurons are involved in environmental enrichment motor protection in a mouse model of Parkinson's Disease. J Mol Neurosci. (2016) 60:453–64. 10.1007/s12031-016-0831-7 27660217

[113] van Praag H Schinder AF Christie BR Toni N Palmer TD Gage FH. Functional neurogenesis in the adult hippocampus. Nature. (2002) 415:1030–4. 10.1038/4151030a 11875571 PMC9284568

[114] Kerr AL Steuer EL Pochtarev V Swain RA. Angiogenesis but not neurogenesis is critical for normal learning and memory acquisition. Neuroscience. (2010) 171:214–26. 10.1016/j.neuroscience.2010.08.008 20804819

[115] Lista I Sorrentino G. Biological mechanisms of physical activity in preventing cognitive decline. Cell Mol Neurobiol. (2010) 30:493–503. 10.1007/s10571-009-9488-x 20041290 PMC11498799

[116] Fredriksson A Stigsdotter IM Hurtig A Ewalds-Kvist B Archer T. Running wheel activity restores MPTP-induced functional deficits. J Neural Transm. (2011) 118:407–20. 10.1007/s00702-010-0474-8 20852902

[117] Chen YH Kuo TT Kao JH Huang EY Hsieh TH Chou YC. Exercise ameliorates motor deficits and improves dopaminergic functions in the rat hemi-Parkinson's model. Sci Rep. (2018) 8:3973. 10.1038/s41598-018-22462-y 29507426 PMC5838260

[118] Tinazzi M Gandolfi M Artusi CA Lanzafame R Zanolin E Ceravolo R. Validity of the wall goniometer as a screening tool to detect postural abnormalities in Parkinson's disease. Parkinsonism Relat Disord. (2019) 69:159–65. 10.1016/j.parkreldis.2019.10.024 31704143

[119] Srivanitchapoom P Hallett M. Camptocormia in Parkinson's disease: definition, epidemiology, pathogenesis treatment modalities. J Neurol Neurosurg Psychiatry. (2016) 87:75–85. 10.1136/jnnp-2014-310049 25896683 PMC5582594

[120] Schlenstedt C Gavriliuc O Boße K Wolke R Granert O Deuschl G. The effect of medication and deep brain stimulation on posture in parkinson's disease. Front Neurol. (2019) 10:1254. 10.3389/fneur.2019.01254 31849818 PMC6901659

[121] Schlenstedt C Boße K Gavriliuc O Wolke R Granert O Deuschl G. Quantitative assessment of posture in healthy controls and patients with Parkinson's disease. Parkinsonism Relat Disord. (2020). 10.1016/j.parkreldis.2020.01.012. [Epub ahead of print]. 32033879

[122] Doherty KM van de Warrenburg BP Peralta MC Silveira-Moriyama L Azulay JP Gershanik OS Postural deformities in Parkinson's disease. Lancet Neurol. (2011) 10:538–49. 10.1016/S1474-4422(11)70067-9 21514890

[123] Fasano A Geroin C Berardelli A Bloem BR Espay AJ Hallett M. Diagnostic criteria for camptocormia in Parkinson's disease: a consensus-based proposal. Park Relat Disord. (2018) 53:53–7. 10.1016/j.parkreldis.2018.04.033 29759930 PMC7293065

[124] Orcioli-Silva D Beretta VS. Applicability of the wall goniometer in Parkinson's disease. Parkinsonism Relat Disord. (2019) 69:157–8. 10.1016/j.parkreldis.2019.11.014 31757617

